# Misalignment Fault Diagnosis for Wind Turbines Based on Information Fusion

**DOI:** 10.3390/e23020243

**Published:** 2021-02-20

**Authors:** Yancai Xiao, Jinyu Xue, Long Zhang, Yujia Wang, Mengdi Li

**Affiliations:** 1 School of Mechanical, Electronic and Control Engineering, Beijing Jiaotong University, Beijing 100044, China; 20126082@bjtu.edu.cn (J.X.); shiyanshi10071@163.com (Y.W.); shiyanshi10072@163.com (M.L.); 2 Department of Electrical and Electronic Engineering, The University of Manchester, Manchester M139PL, UK; long.zhang@manchester.ac.uk

**Keywords:** wind turbines, misalignment, fault diagnosis, information fusion, improved artificial bee colony algorithm, LSSVM, D–S evidence theory

## Abstract

Most conventional wind turbine fault diagnosis techniques only use a single type of signal as fault feature and their performance could be limited to such signal characteristics. In this paper, multiple types of signals including vibration, temperature, and stator current are used simultaneously for wind turbine misalignment diagnosis. The model is constructed by integrated methods based on Dempster–Shafer (D–S) evidence theory. First, the time domain, frequency domain, and time–frequency domain features of the collected vibration, temperature, and stator current signal are respectively taken as the inputs of the least square support vector machine (LSSVM). Then, the LSSVM outputs the posterior probabilities of the normal, parallel misalignment, angular misalignment, and integrated misalignment of the transmission systems. The posterior probabilities are used as the basic probabilities of the evidence fusion, and the fault diagnosis is completed according to the D–S synthesis and decision rules. Considering the correlation between the inputs, the vibration and current feature vectors’ dimensionalities are reduced by t-distributed stochastic neighbor embedding (t-SNE), and the improved artificial bee colony algorithm is used to optimize the parameters of the LSSVM. The results of the simulation and experimental platform demonstrate the accuracy of the proposed model and its superiority compared with other models.

## 1. Introduction

In order to address global warming issues, many countries have reduced carbon emissions year by year as one of their targets for economic and social development. As one typical source of clean energy, wind power has significant advantages in terms of environmental and ecological impact compared with hydropower and nuclear power [1]. In recent years, wind power has been rapidly developed in many countries, and the installed capacity has been increasing year by year [2].

The working environment of wind turbines is often complex, so the failure rate of the components of wind turbines is relatively high [3]. If the key components of the wind turbine system fail, it will cause damage and even stop the whole turbine, resulting in huge economic losses. Therefore, in recent years, a large number of research work has been focused on fault diagnosis of wind turbines. The failures typically include blade failures, transmission system failures, generator failures, and tower failures. Among them, misalignment of the transmission system is one of the common failures [4]. Many reasons, such as bearing eccentricity, installation error, and coupling misalignment, can cause misalignment of the wind turbine transmission system that connects the gearbox and generator for a typical doubly-fed wind turbine [5]. The misalignment of the transmission system can inevitably lead to vibration of the unit, which will reduce the reliability of the power generation system. In addition, the misalignment failure can cause damage to gears and bearings [6]. Therefore, it is necessary to monitor and diagnose the misalignment of the transmission system in doubly-fed wind turbines.

Although there is much work on the misalignment fault diagnosis for a conventional rotating system, there is little work for wind turbine misalignment diagnosis. In particular, a wind turbine presents additional and unique challenges as it operates under variable rotational conditions [7,8]. At present, the main research on detecting the misalignment of wind turbines includes the following work. Zhao et al. applied variational mode decomposition (VMD) to decompose the fault vibration signal to isolate features and diagnose the misalignment faults in a direct drive wind turbine [9]. Abdalla et al. diagnosed misalignment of planetary gearbox based on vibration measurements using spectrum analysis and modulation signal bispectrum (MSB) analysis [10]. Huang et al. applied the Hilbert–Huang transform (HHT) method for fault diagnosis of wind turbine rotors and discussed three typical faults by the HHT, including rotor mass imbalance, aerodynamic asymmetries, and yaw misalignment [11]. An and Kong proposed a modified empirical mode decomposition (EMD) method to extract characteristics from vibration signals and applied a back-propagation neural network to data from various sensors to diagnose faults of offshore wind turbines included stator imbalanced, rotor unbalanced, and bearing misalignment [12]. Villa et al. developed a statistical diagnosis algorithm based on the significance level of the modeled fault to detected unbalance fault and misalignment fault of wind turbine, and tested the algorithm on vibration from a test-bed [13]. He et al. analyzed the vibration characteristics of the transmission chain of a wind turbine based on double-elastic support with natural axial misalignment between the output shaft of gearbox and the shaft of generator causing vibration signals of normal gearbox blend with serious high-order gear mesh frequency and smooth modulation [14]. However, these methods mainly applied rely on single information, and their performance could be limited owing to the limited source of information.

Because the diagnosis based on single information often cannot reflect the overall condition, the information fusion methodology for multiple source information is needed for the diagnostic system. Information fusion is a synchronous and comprehensive processing of the information obtained from multiple sensors. It can ensure the integrity of the information from a different perspective and overcome the shortcomings of traditional single information to form a more objective and closer understanding of the system [15], which can greatly improve the accuracy of diagnosis.

Information fusion can be divided into three levels: data level, feature level, and decision level [16,17].

- Data level fusion. The direct fusion of signals collected by the same type of sensors retains the most information among the three levels.
- Feature level fusion. In this process, the signals from multiple sensors need to be preprocessed. Features are extracted to form the fusion vector and its attributes are used to judge the state of targets to be diagnosed.
- Decision level fusion. After initial state judgment of the target to be diagnosed, the final state is obtained based on the fusion of some decision rules. Decision level fusion is the highest among the three levels. Its real-time performance and fault tolerance are very good, but the information loss is very large, so more complex algorithms are needed.

At present, there are many research methods and achievements in decision level fusion, including Bayesian theory [18], Dempster–Shafer (D–S) evidence theory [19], fuzzy set theory [20,21], rough set theory [22], and so on. The classification principle of Bayesian theory is to calculate the posterior probability of an object (the probability that the object belongs to a certain class) using the prior probability and Bayes formula, and select the class with the largest posterior probability as the one to which the object belongs. In D–S evidence theory, trust function and likelihood function are obtained by calculating the orthogonal sum of basic probability distribution functions of different evidences. After fusing multiple evidences, the final decision is made according to decision rules. Among them, basic probability distribution function is the probability distribution of all possible faults in each state, trust function is the lower bound of fault event probability, and likelihood function is the upper bound. Fuzzy set theory (FS) was founded by Zadeh. Membership T(x) was used to describe fuzzy information. At this time, non-membership F(x) did not appear. Then, intuitionistic fuzzy sets (IFSs) and interval intuitionistic fuzzy sets (IVIFSs) appeared successively. The fuzzy information processing technology developed from fuzzy set theory can provide a simple and effective means to explore uncertainty and simulate human recognition mechanism. Rough set theory, initially developed by Pawlak (1982), is a mathematical tool that deals with vague, uncertain, and incomplete information. Rough set theory has been successfully applied in many fields such as machine learning, pattern recognition, control systems, data mining, and image classification.

The advantages and limitations of the above four methods are listed in Table 1.

In this paper, based on the good theoretical basis and application effect of D–S evidence theory [23,24,25,26,27], it is used to complete decision fusion, which provides a sufficient fault diagnosis solution for wind turbine misalignment fault.

The aim of this paper is to use multiple sources of information to distinguish the misalignment-free (normal condition) and three different types of transmission misalignment. The main contributions are summarized as follows.

Multiple sources of information and integrated approach are used for wind turbine transmission misalignment detection. More specifically, the vibration, temperature, and stator current signal are taken as the original source, and their time domain features, frequency domain features, and time-frequency domain features are extracted as fault characteristics. t-distributed stochastic neighbor embedding (t-SNE) is used to reduce the vibration and current characteristics dimensionality, and then three posterior probability least squares support vector machine with parameters optimized by improved artificial bee colony algorithm are constructed. The probability outputs of the three LSSVM are taken as the basic probabilities of evidence fusion. The probability distribution after fusion is calculated according to the Dempster fusion rule. Compared with the non-fusion models, it is demonstrated that the model based on D–S evidence fusion has higher diagnostic accuracy for wind turbine misalignment faults.

The remainder of the paper is organized in the following way. In Section 2, the formulas of D–S evidence theory, posterior probability least squares support vector machines, and the improved artificial bee colony are presented in detail. Section 3 describes the specific steps for D–S fault diagnosis. Section 4 presents the fault diagnosis case study based on the simulation model. Section 5 presents the fault diagnosis case study based on the experimental platform. Section 6 concludes the current work.

## 2. Theoretical Background

### 2.1. D–S Evidence Theory

The D–S evidence theory is a method of uncertainty reasoning, proposed by Dempster in 1967 and later improved and developed by Shafer [28]. The D–S evidence method can produce a probability interval to an uncertain event by fusing multiple evidences with known probability distribution. As an indeterminate reasoning method, D–S evidence theory uses weaker conditions than Bayesian, and has the ability to quantify unknown and uncertainty [29]. The evidence theory contains three important functions: basic probability assignment function, belief function, and plausibility function. The basic probability assignment function is the probability distribution of all possible faults in each state, the belief function is the lower bound of the probability of the fault event, and the plausibility function is the upper bound of the probability of the fault event. The belief function and the plausibility function can be obtained by calculating the sum of the basic probability assignment function, and the final decision is made after combining multiple evidences from different sources.

The D–S evidence theory consists of the following parts [30].

- Frame of discernment:

A variety of possible mutually exclusive hypothesis $X_{i}\left(i=1,2,\cdots ,s\right)$ of a question constitute a finite and non-empty set, which is called the frame of discernment, denoted as $\Omega =\left\{X_{1},X_{2},\cdots ,X_{s}\right\}$.

- Basic probability assignment (BPA) function:

BPA function is also known as the mass function. Suppose H is a subset of Ω, if function m(H) satisfying

$$\begin{array}{c} (a)\;m\left(\phi \right)=0\\ (b)\;\sum m\left(H\right)=1\\ (c)\;m\left(H\right)>0 \end{array}$$

then, function m(H) is called the basic probability assignment of H on Ω.

- Belief function:

In the frame of discernment, the belief function represents the sum of the basic probability assignment functions of all subsets of H. The expression of the belief function is as follows:

(1) $$bel\left(H\right)=\sum_{Y∕Y\subseteq H}m\left(Y\right)$$

- Plausibility function:

In the frame of discernment, the plausibility function represents the degrees of belief for not denying H, which is the sum of the basic probability assignments of all the subsets intersecting H. The expression of the plausibility function is as follows:

(2) $$pl\left(H\right)=\sum_{S/S\cap H\neq \emptyset }m\left(S\right)$$

- Dempster’s rule of combination:

Dempster’s rule of combination is used to combine the BPA functions of multiple evidences. Although this rule is controversial at present, the authors of [31] have showed that it behaves perfectly when evidences do not conflict reciprocally. Only if we integrate conflicting evidences do we need to improve it. In this paper, there is no serious and complete conflict among the outputs from vibration signal, temperature signal, and stator current signal as evidences in this study. Therefore, Dempster’s rule is still used here.

Suppose there are n independent evidences (sensors or expert opinions), $H_{1},H_{2},\cdots ,H_{n}$ (are subsets of  Ω), the BPA of them are $m_{1},m_{2},\cdots ,m_{n}$. Then, Dempster’s rule for the BPA functions on Ω is as follows:

(3) $$m=m_{1}⊕m_{2}⊕\cdots ⊕m_{n}$$

Specifically, it can be expressed as follows:

(4) $$m\left(H\right)=\frac{\sum_{H_{1}\cap \cdots \cap H_{n}}m_{1}\left(H_{1}\right)m_{2}\left(H_{2}\right)\cdots m_{n}\left(H_{n}\right)}{1-K},H\neq \emptyset$$

where the expression of K is as follows:

(5) $$K=\underset{H_{1}\cap \cdots \cap H_{n}=\emptyset }{\sum }m_{1}\left(H_{1}\right)m_{2}\left(H_{2}\right)\cdots m_{n}\left(H_{n}\right)$$

where *K* is the degree of conflict between evidences. When *K* = 1, the evidences are completely conflicted and cannot be synthesized by this formula; when *K* tends to 1, the evidences are highly conflicted, and synthesizing by this formula may lead to results contrary to fact [32].

**Figure 1:**
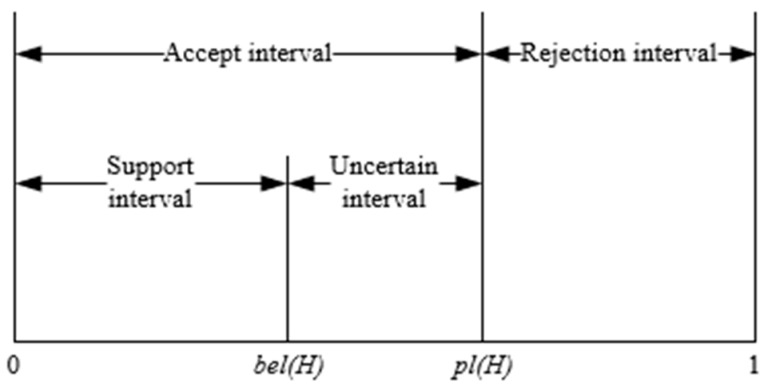
Uncertainty representation of a proposition.

- Decision rules:

The decision rule is to draw a diagnosis based on the uncertain interval [bel(H),pl(H)] of the evidence. In the interval of [0,1], the uncertainty of a proposition is shown in Figure 1.

In Figure 1, [0,bel(H)] belongs to the support interval, [0,pl(H)] is the accept interval, [pl(H),1] is the rejection interval, and [bel(H),pl(H)] is the uncertain interval.

When making a decision, choose a value in the uncertain interval as the final trustworthiness of the proposition. If this value has the highest trustworthiness among the possible hypothesis, this assumption is the final decision result.

### 2.2. Posterior Probability Least Squares Support Vector Machine

In the study, the fault samples collected are limited, while support vector machine (SVM) and LSSVM can obtain high diagnosis accuracy based on small sample data. Moreover, the speed of LSSVM is faster than that of SVM, so LSSVM is selected to be the initial classifier to judge the state. As the input parameters of the D–S evidence fusion are basic probability assignments in all classification spaces, the hard output (whether or not) of the traditional classifier has to be converted to a soft one (probability) [33]; that is, the output of the classifier must be changed to the posterior probability output. For the two-class problem, the posterior probability can be calculated using the sigmoid function to map the output f(x) (+1,−1) of the LSSVM to the [0,1] interval. Assuming that the probability is consistent with the sigmoid distribution, the posterior probability can be calculated [34]:

(6) $$p\left(y=1/f\right)=\frac{1}{1+exp\left(Af+B\right)}$$

(7) p(y=−1/f)=1−p(y=1/f)

where f is the classification result of the standard LSSVM, p(y=1/f) is the probability when the classification is correct under the condition that the output value is f, p(y=−1/f) is the probability when the classification is wrong under the condition that the output value is f, and A and B are parameters. So, the key to calculating the posterior probability is to obtain parameters A and B. The posterior probability least squares support vector machine model is usually established by first establishing a standard LSSVM model, and then obtaining A and B on the training set $\left(f_{i},t_{i}\right)$, where $t_{i}$ is the target probability output of the standard LSSVM:

(8) $$t_{i}=\{\begin{array}{c} \frac{N_{+}+1}{N_{+}+2},f_{i}=+1\\ \frac{1}{N_{-}+2},f_{i}=-1 \end{array}$$

where $N_{+}$ is the number of positive samples; $N_{-}$ is the number of negative samples; and the problem of obtaining parameters A and B is to solve the minimum likelihood optimization problem of the following, i.e.,

(9) $$min\left\{-\overset{n}{\underset{i=1}{\sum }}\left[t_{i}\log\left(p_{i}\right)+\left(1-t_{i}\right)log\left(1-p_{i}\right)\right]\right\}$$

where

(10) $$p_{i}=\frac{1}{1+exp\left(Af_{i}+B\right)}$$

The Hessian matrix for solving (9) is as follows:

(11) $$H\left(z\right)=\left[\begin{array}{cc} \underset{i}{\sum }f_{i}{}^{2}p_{i}\left(1-p_{i}\right) & \underset{i}{\sum }f_{i}p_{i}\left(1-p_{i}\right)\\ \underset{i}{\sum }f_{i}p_{i}\left(1-p_{i}\right) & \underset{i}{\sum }p_{i}\left(1-p_{i}\right) \end{array}\right]$$

In order to get the minimum value of (9), the Hessian matrix must be positively determined. So, A and B are finally obtained by solving all the eigenvalues of the matrix that are greater than zero. The posterior probability can be obtained.

It is proved that the posterior probability least squares support vector machine with sigmoid function works well in practical applications [35], but this method can only be used for the two-class problem. The main methods for extending LSSVM from two-class to multi-class are the “one-versus-one” and “one-versus-all” methods. The Platt algorithm calculates the probability formula for each classifier as follows, where $p_{m}$ is the probability that sample x belongs to the i-th class [35]:

(12) $$p_{m}=\left(y=m/x\right)=\frac{1}{1+exp\left(A_{m}f\left(x\right)+B_{m}\right)}$$

### 2.3. The Improved Artificial Bee Colony

There are three kinds of kernel functions commonly used in LSSVM: linear kernel function, polynomial kernel function, and radial basis function (RBF) ($K\left(x_{i},x_{i}\right)=exp\left(-∥x_{i}-x_{i}{∥}^{2}/\sigma^{2}\right)$*,* where σ is the kernel width). Many studies and experiments [36] show that, compared with other kernel functions, RBF can map the original space into an infinite dimensional space and find the hyperplane better. It is a better choice as the kernel function. Therefore, it is necessary to select the regularization parameter γ (necessary for LSSVM, determining the trade-off between the training error minimization and smoothness) and the kernel squared bandwidth $\sigma^{2}$.

Choosing a better parameter value can greatly improve the performance of the LSSVM classifier and the accuracy of diagnosis. At present, the commonly used methods include trial and error, cross validation, grid search, and intelligent optimization algorithm [37]. Among them, the trial and error method not only consumes time and energy, but also the choice of parameters is greatly affected by subjective factors; the cross validation method divides the data set into training, validation, and testing, and different proportions will lead to different optimal models and optimal parameters; and the grid search method optimizes the model according to the set step size in the upper and lower limits of parameters, and then determines the optimal parameters, so the search speed is too slow and the precision is not high. Therefore, the advantages of the intelligent optimization algorithm are highlighted. It realizes the optimal distribution of food by simulating the behavior of animals in the population (interact information and cooperation among individuals). A swarm intelligence optimization algorithm is easy to implement and has high efficiency, so it is applied to the parameter optimization process of LSSVM.

Swarm intelligence optimization algorithms include genetic algorithm, particle swarm optimization, artificial fish swarm algorithm, artificial bee colony algorithm, and so on. Among them, artificial bee colony algorithm (ABC) is an optimization algorithm proposed in recent years, which not only has good optimization ability, but also controls less parameters in the process. Furthermore, it is simple, flexible, and easier to implement. The research [38] shows that the optimization performance of ABC is better than that of genetic algorithm and particle swarm algorithm, and the classification diagnosis accuracy of LSSVM optimized by ABC is higher than that of LSSVM optimized by genetic algorithm and particle swarm algorithm.

However, ABC has some shortcomings, such as slow convergence speed in the later stage of operation and the fact that it is easy to fall into local optimum. Therefore, in this paper, on the one hand, chaotic initialization is introduced in the artificial bee colony algorithm, which is used to initialize the population position to improve the diversity of the population and the ergodicity of the population search process. On the other hand, in the collecting bees stage of the artificial bee colony algorithm, the bees are divided into two parts: one part collects the optimal information of the region according to the original algorithm, and the other does Lévy flight around the global optimal solution to improve their global search capabilities. At the same time, in the observing bees stage, a search strategy based on the current local optimal solution (called pbest) is adopted to improve the local search ability of the algorithm.

(1) The logistic chaotic map is proposed to initialize the population. The equation for the logistic chaotic map is as follows:

(13) $$y_{t+1}=\mu y_{t}\left(1-y_{t}\right)\;t=0,1,\cdots ,l$$

In the formula,$\;y_{t}\in \left(0,1\right)$, t is the number of iterations of the chaotic sequence, μ is the control parameter of the chaotic sequence, and the value range is [3.75,4] [39].

(2) Lévy flight was introduced in the evolution strategy to improve the performance of the algorithm and achieve good results [39]. The calculation method is based on

(14) $$L\left(\alpha \right)={\left[\frac{\Gamma \left(1+\alpha \right)sin\left(\frac{\pi \alpha }{2}\right)}{\Gamma \left(\frac{1+\alpha }{2}\right)\alpha 2^{\left(\frac{\alpha -1}{2}\right)}}\right]}^{1/\alpha }$$

where α is the characteristic index, which usually satisfies 0<α<2. Γ(·) is the Gamma function defined as

$$\Gamma \left(z\right)=\int_{0}^{\infty }t^{z-1}e^{-t}dt.$$

Its update equation is as follows:

(15) $$v_{ij}=x_{ij}+\alpha \left(x_{ij}-x_{best}\right)L\left(\alpha \right)$$

where α is the step length, which usually meets the standard normal distribution, and L(·) is the random search path for Lévy flight.

(3) In the observing bees stage, for any current solution in each generation, the top p% solutions are randomly selected among all current solutions, and the best one (called pbest) can be used to balance global search capabilities and local development capabilities. The neighborhood search formula is as follows:

(16) $$v_{ij}=x_{ij}+\varphi_{ij}\left(x_{ij}-x_{kj}\right)+\emptyset_{ij}\left(x_{randp,j}^{best}-x_{ij}\right)$$

where $\;k\in \left\{1,2,\cdots ,S_{N}\right\}$, $S_{N}$ is the number of solutions for the bee colony, j∈{1,2,⋯D}, D is the dimension of the optimization problem, k≠i, $\varphi_{ij}\in \left[-1,1\right]$, and $\emptyset_{ij}\in \left[0,1.5\right]$.

## 3. Specific Steps for Misalignment Diagnosis

D–S evidence theory is used to carry out the fault diagnosis of wind turbines. The specific steps are as follows.

(1) Identify the frame of discernment of the fault diagnosis system

The frame of discernment is the common faults of the wind turbines misalignment in the study. At the same time, the normal working state of the unit is added. So, the frame of discernment is expressed as follows: {normal, parallel misalignment, angular misalignment and integrated misalignment}.

(2) Determination of evidence

The posterior probability least squares support vector machines are trained by the vibration signal, the temperature signal, and the stator current signal feature vectors separately. The hard outputs of the traditional LSSVM are mapped to the [0,1] interval using the sigmoid function. The soft outputs of the transformation are used as evidences for D–S evidence theory.

(3) Determination of basic probability assignment function, belief function, and plausibility function

The three least squares support vector machines give the probability vectors of all the classifications on the entire identification framework respectively, and the probability vectors to be directly used as the basic probability assignments, belief function, and plausibility function can be obtained by calculation.

(4) Evidence synthesis and diagnosis

According to Dempster’s law, the probability vectors directly participate in the evidence fusion process. After the final probability vector is given, the final diagnosis result based on the probability vector after fusion can be obtained.

**Figure 2:**
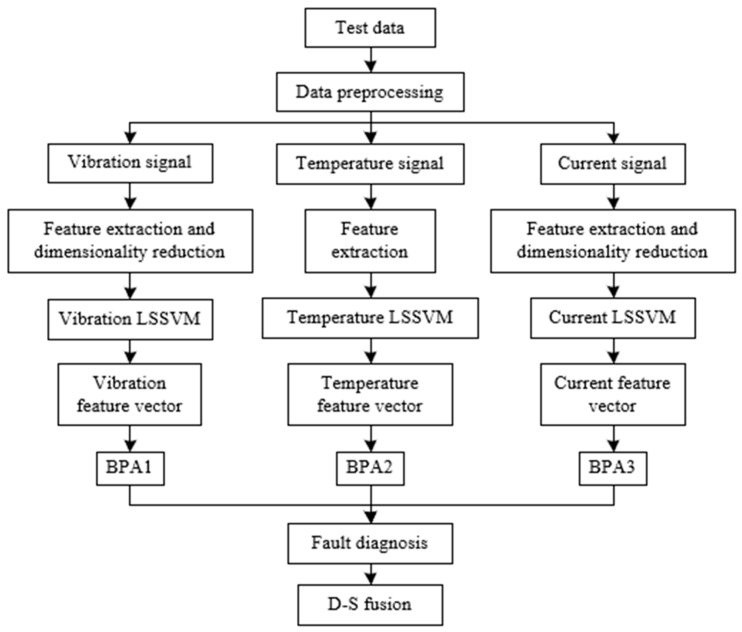
Process of Dempster–Shafer (D–S) evidence fusion. LSSVM, least square support vector machine; BPA, basic probability assignment.

Figure 2 summarizes process of D–S evidence-based misalignment diagnosis.

## 4. The Simulation Case Studies of Misalignment Fault Diagnosis

The simulation wind turbine system is established by ADAMS 2013, MATLAB R2014a, and Ansys 17.0. The three-dimensional (3D) model of the 1.5 MW wind turbine is established using SolidWorks, and then it is imported into ADAMS 2013, where the Marker point is moved according to the type and degree of misalignment; that is, parallel misalignment is simulated by making the center of mass deviate from the center of rotation for a certain distance; angle misalignment is simulated by rotating the marker a certain angle around the y-axis, and placing the rotation axis of the coupling relative to the ground on the z-axis of the Marker point; and integrated misalignment is simulated by adding the parallel misalignment and angle misalignment in the local coordinate system (maker) of the left half coupling at the same time. The correctness of the models has been verified in the literature [40]. The vibration signals were extracted under the input speeds of 81.3°/s, using step function as the input of ADAMS, the simulation time is 1.5 s, and simulation steps are 6000 steps. The wind turbine models and its control system are established by SIMULINK/MATLAB, where the stator current was sampled at the same speed at which the vibration signal was sampled, and the sample frequency is 200 kHz. The correctness of the models has been verified in the literature [41]. After that, the high-speed gear shaft and the main shaft of the generator are introduced into HyperMesh to divide the grid. Then, the model is imported into Ansys Workbench to get the corresponding temperature signals (details in the literature [42]). In this paper, 100 samples are taken for each of the four types of diagnostic states (normal, parallel misalignment, angular misalignment, and integrated misalignment), of which 60 are for training and 40 are for testing. So, there are 240 (60 × 4) samples in the training set and there are 160 (40 × 4) samples in the testing set.

### 4.1. Data Processing

After the vibration signal, temperature signal, and stator current signal under four working conditions are collected, in order to make better use of them and get good diagnosis results, the feature indexes in the time, frequency, and time–frequency domain are extracted. Table 2 shows a 21-dimension mixed feature library of the vibration signal.

Suppose signal *x* ($x_{0},\;x_{1},\;x_{2},\cdots ,\;x_{N-1}$) is a discrete time series with a finite length, the calculation formulas of time domain characteristic indexes are shown in Table 3, where $\bar{x}$ is the mean value of the signal, $x^{\prime }$ is the average amplitude, and $x_{p}$ is the peak value of the signal.

In signal analysis, power spectrum analysis is usually used to extract the frequency domain index. Center of gravity frequency, mean square frequency, root mean square frequency, and frequency variance are commonly used. The sampling frequency is set as $f_{s}$, and the calculation formula of each index is shown in Table 4, where S(ω) is the power spectrum of discrete time series, $S\left(\omega \right)=X\left(\omega \right)\cdot \bar{X\left(\omega \right)}$, $X\left(\omega \right)=\sum_{i=0}^{N-1}x\left(i\right)e^{-j\pi \omega },\;\omega \;$ is the angular frequency.

Time–frequency analysis is a fault diagnosis method that combines the law and reason of frequency changing with time. In this paper, image extended empirical mode decomposition (IEMD) is used to process the vibration signal, and dual tree complex wavelet transform (DTCWT) is used to process the stator current signal (see the literature [43] for details).

The gearbox tooth temperature $T_{1}$ and the generator rotor shaft temperature $T_{2}$ are selected as the characteristic values of the temperature signal. Construct a two-dimensional vector of the temperature signal: $X=\left[T_{1},T_{2}\right]$.

Table 5 is a mixed feature library with a total of 29 dimensions in the time domain, frequency domain, and time–frequency domain of the stator current signal (see the literature [41] for details).

In order to eliminate the influence of different input dataset dimensions and large numerical differences, the original dataset is normalized, i.e.,

(17) $$y=\frac{y_{max}-y_{min}}{x_{max}-x_{min}}*\left(x-x_{\min}\right)+y_{min}$$

where x is the value to be normalized, $y_{min}$ is the lower bound of the normalized interval, and $y_{max}$ is the upper bound of the normalized interval. In this paper, $y_{min}=0$, $y_{max}=1$, and the vector is normalized by column.

Because of the high dimensionality of the constructed vectors of the vibration signal and the stator current signal, not only does the amount of calculation increase, but also some difficulties are brought to fault diagnosis [44]. In order to make better use of various information and obtain good diagnostic results, the feature vectors are subjected to dimensionality reduction using t-SNE.

t-SNE based on conditional probability retains the similarity between high-dimensional and low dimensional space data and adopts symmetric objective function, and t distribution in low-dimensional space replaces Gaussian distribution, which solves the problem of crowding and clear visualization in low-dimensional space [45]. Its implementation steps are as follows:

(1) Define a high-dimensional data set: $x=\left\{x_{1},x_{2},\cdots ,x_{n}\right\}$.

(2) Compute the complexity parameter of the value equation c:

(18) $$c=\sum_{i}\sum_{j}p_{ij}log\frac{p_{ij}}{q_{ij}}$$

(19) $$perp\left(p_{i}\right)=2^{H\left(p_{i}\right)}$$

(20) $$H\left(p_{i}\right)=-\sum_{j}p_{j∕i}log_{2}^{p_{j∕i}}$$

where $p_{i}$ is the conditional probability of data points (other than $x_{i}$) with respect to $x_{i}$, $p_{j∕i}$ is the conditional probability of high-dimensional data, $p_{ij}$ is the joint probability density in the high-dimensional space, and $q_{ij}$ is the joint probability density in the low-dimensional mapping space.

(3) Define the optimization parameters: the number of iterations T, the learning rate η, and the momentum factor at the tth (t≤T) iteration α(t) (0<α(t)<1). The value equation c is learned by the gradient descent method, and the low-dimensional mapping of the high-dimensional data is finally obtained:

(21) $$\frac{\delta c}{\delta y_{i}}=4\sum_{j}\left(p_{ij}-q_{ij}\right)\left(y_{i}-y_{j}\right){\left(1+∥y_{i}-y_{j}{∥}^{2}\right)}^{-1}$$

where $y_{i}$ and $y_{j}$ are the mapping of the high-dimensional data $x_{i}$ and $x_{j}$ in the low-dimensional space.

In order to speed up the optimization process and prevent trapping into local minima, a relatively large momentum condition is imposed on the descent process. The current gradient value is summed to the previous gradient value for each iteration and then decays exponentially to determine the coordinates of the low-dimensional data. The momentum formula is as follows:

(22) $$y^{\left(t\right)}=y^{\left(t-1\right)}+\eta \frac{\delta c}{\delta y}+\alpha \left(t\right)\left(y^{\left(t-1\right)}-y^{\left(t-2\right)}\right)$$

where y is the data in the low-dimensional space.

### 4.2. The Fault Diagnosis Results

In this paper, “one-versus-all” is used to extend LSSVM from two classifications to multiple classifications. That is, each time, one fault is selected as one type, and the rest of the states are selected as another type. In order to produce the posterior probabilities of the four classifications in the vibration feature space, four two-class LSSVM are constructed, and each LSSVM calculates a set of A and B, and then the corresponding posterior probability is calculated according to (5) and (6). In the same way, the probability vectors of the temperature and stator current signal classifiers for the four states can be obtained as the BPA of D–S evidence fusion.

The five-dimensional feature vectors of the vibration signal after t-SNE dimensionality reduction are used as the inputs, and the four working conditions of the transmission system are used as outputs to train the LSSVM, which is optimized by the improved artificial bee colony algorithm. The parameters of the four two-classification LSSVM in the vibration feature space are shown in Table 6. Four samples are selected, such as samples 5, 44, 82, and 130, and the corresponding BPA1 calculated is shown in Table 7.

The two-dimensional feature vectors of the temperature signal are used as the inputs, and the four operating states of the transmission system are used as the outputs to train the optimized LSSVM. The parameters of the four binary LSSVM in the temperature feature space are shown in Table 8. The BPA2 calculated from the same four samples is shown in Table 9.

The four-dimensional vectors after the dimensionality reduction of the stator current signal are used as inputs, and the four operating states of the transmission system are as outputs to train the optimized LSSVM. The parameters of the four two-class LSSVM in the stator current feature space are shown in Table 10, and the BPA3 calculated by the same four samples is shown in Table 11.

**Figure 3:**
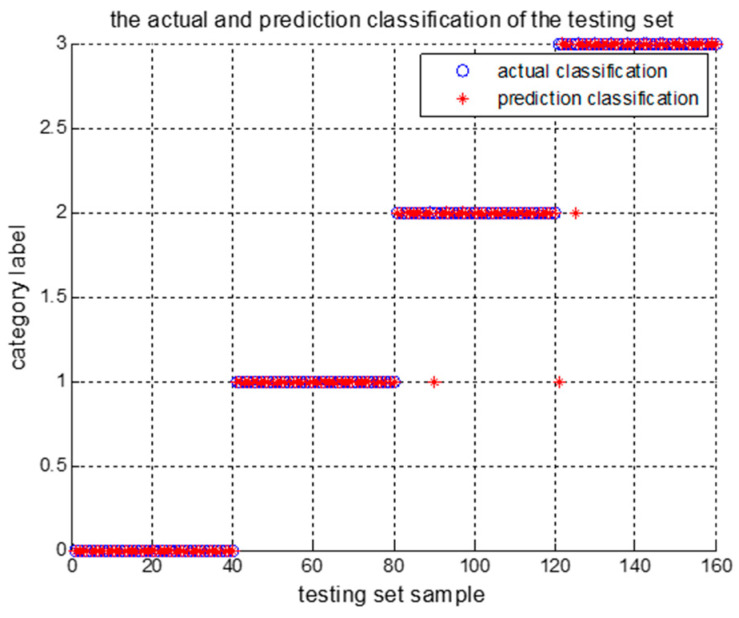
The diagnosis results of the testing set.

Then, the probability assignments are calculated after the fusion of the three BPAs. The category with the highest degree of belief is selected as belonging to the class of the fusion model. Table 12 shows the basic and the fusion probability of the three LSSVM outputs for the selected test samples. Table 13 shows the fusion and classification results of the four test samples. Figure 3 shows the test samples’ diagnosis results, in which “0” indicates normal operation, “1” indicates parallel misalignment, “2” indicates angular misalignment, and “3” indicates integrated misalignment.

In order to better evaluate the performance of the fault diagnosis method, three indexes are adopted: the training set classification accuracy, the testing set classification accuracy, and the fault false alarm rate. The fault false alarm rate means that the fault does not actually occur, but the fault detection alarm is given by the detection system. The false alarm rate equals the number of false alarm samples divided by the total number of actual fault-free samples. Table 14 compares the results of the sample sets diagnosed by the indexes of a single signal (vibration, temperature, or current signal) with the D–S evidence fusion.

From Table 14, it can be seen that the accuracy of D–S fusion is higher than that of any single signal, and the failure false alarm rate is equal to zero, lower than others, which proves the advantage of information fusion in the diagnosis of wind turbine misalignment fault.

## 5. Experimental Verification of Platform

**Figure 4:**
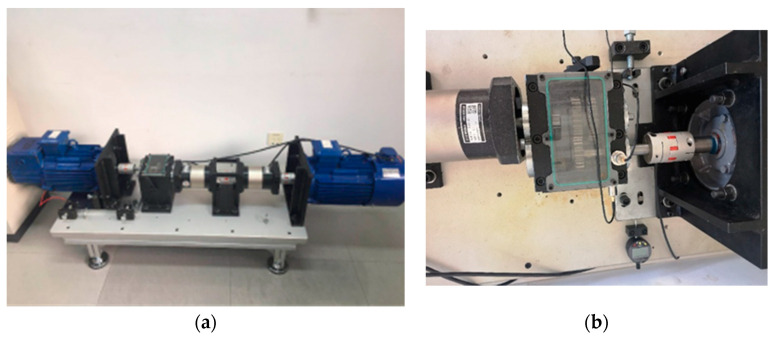

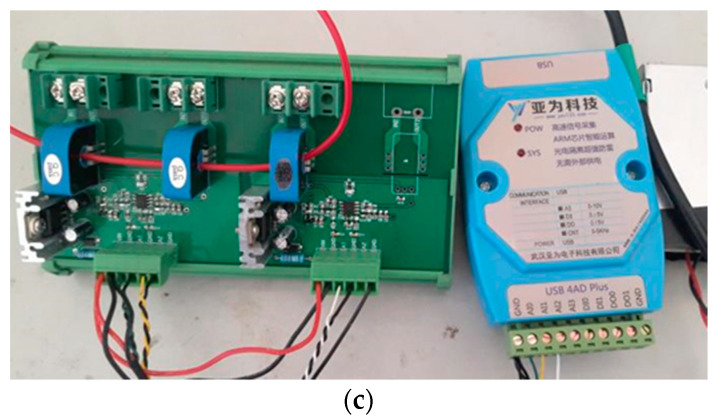
Experiment equipment. (**a**) The platform of wind turbine; (**b**) layout of vibration sensor; (**c**) current signal acquisition card USB 4AD Plus.

In this paper, the 1.5 kW misalignment experimental platform is used for experimental verification. The platform is shown in Figure 4a. It includes a generator, coupling, gearbox, driving motor, and so on. The speed of the driving motor is changed by a planetary gear reducer with a transmission ratio of 1:50 to simulate the wind blowing blade speed, then it is accelerated by a planetary gear with a transmission ratio of 40:1 and a spur gear with a transmission ratio of 1.5:1 to drive the generator. The generator can be adjusted by the support to create parallel or angular misalignment.

**Figure 5:**
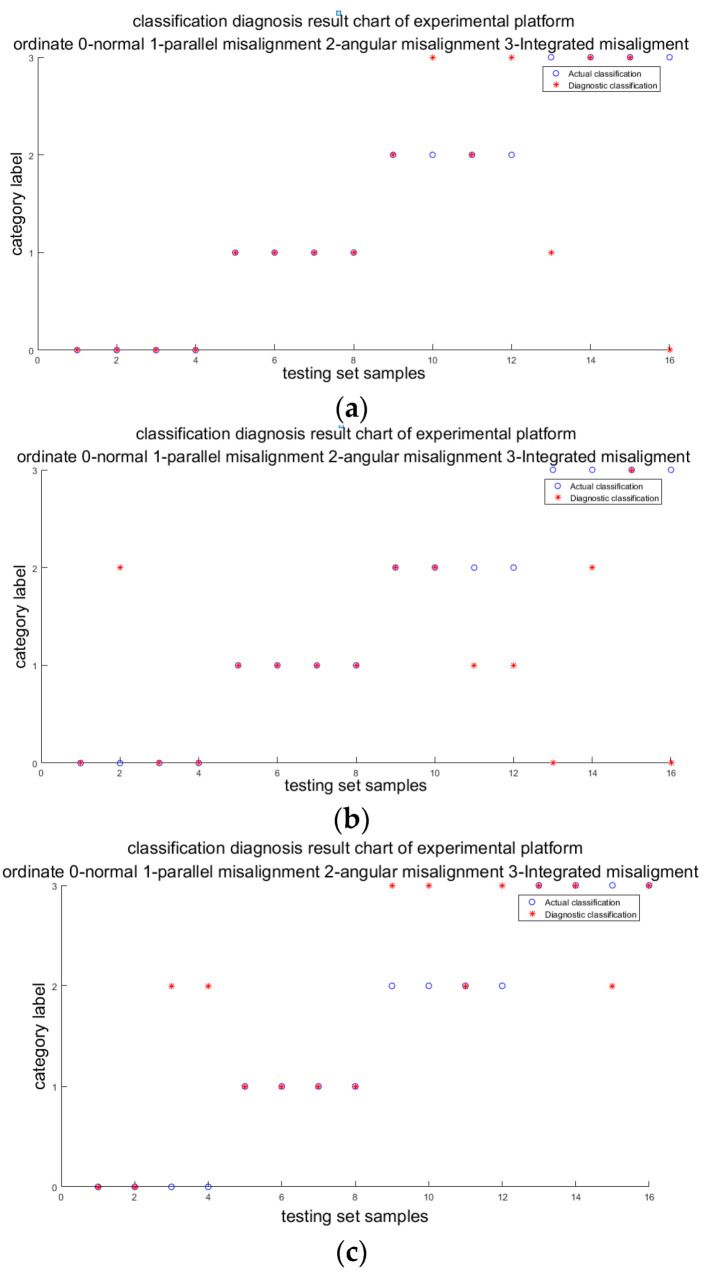
Diagnostic result. (**a**) Vibration signal + current signal; (**b**) vibration signal; (**c**) current signal.

The vibration signal of the gearbox is obtained using the DFT5100 dynamic data collector from the acceleration sensor (ICP type) on the experimental platform (Figure 4b). The current signal is transmitted to the USB signal acquisition and recording platform through the signal acquisition card USB 4AD Plus (Figure 4c). In this paper, the rotation speed of the motor is set to 600 rpm; the sampling time is 10 s; and the sampling frequency of vibration and current is 1 kHz and 2 kHz, respectively. In the experiments, the temperature signal is easily affected by the operation time of the unit and the ambient temperature, and it cannot reflect the actual operating temperature of the wind turbine. Therefore, when fusing different signals by D–S evidence theory, we set the temperature signal to 0, regardless of its influence. Four groups for each working condition, with a total of 16 groups, are sampled on the platform. Some characteristic indexes of vibration and current signal are shown in Table 15 and Table 16. The actual classification and diagnosis results of fusion signals and individual signals are shown in Figure 5. Table 17 is the calculation of two examples.

It can be seen from Figure 5 that the classification accuracy of the testing set is 75%, while that of the single vibration signal is 62.5% and that of the single current signal is 62.5%, which indicates that the accuracy of the diagnosis is improved by using the D–S decision fusion method with multi-source signals as the diagnosis information. In addition, the reason the classification accuracy of the experimental results is much lower than that of the simulation results is that there is no temperature signal in the D–S evidence theory fusion. It can be seen from Table 17 that the first sample is correctly identified using either the single signal or fusion signal, while the second sample is mistakenly diagnosed as angle misalignment using only the vibration signal, but is correctly identified by D–S fusion.

## 6. Conclusions

This paper proposes an integrated fault diagnosis method for wind turbine transmission system misalignment based on information decision fusion. The method uses multiple sources of signal including vibration signal, temperature signal, and stator current signal as the original source, and extracts different features from their time domain, frequency domain, and time–frequency domain. t-SNE is used to eliminate the correlation of characteristic values of the vibration signal and the stator current signal. Three posterior probability least squares support vector machines optimized using improved artificial bee colony algorithm are constructed respectively. The output probabilities of least squares support vector machines are used as the basic probability distribution of evidence fusion, and the fault diagnosis is completed by D–S synthesis and decision rules. Finally, the simulation experiments and platform verification show that the D–S evidence fusion model has higher diagnostic accuracy than the non-fusion model for the wind turbine misalignment fault.

## Figures and Tables

**Table 1:** Comparison of information fusion algorithms. D–S, Dempster–Shafer.

Approach	Advantages	Disadvantages
Bayes’ theorem	Takes probability as the input data, has sufficient theoretical knowledge	Difficult to define prior probability function, lacks the ability to allocate the total uncertainty
D–S evidence theory	The premise is easier to meet, no need to know prior probability	Cannot solve the serious conflict or complete conflict of evidences
Fuzzy set theory	Based on local theory of classification, has strong adaptive ability	Determines the uncertainty according to the subjective judgement
Rough set theory	Deals with redundant information and inconsistent information effectively	Discretization of symptom attributes is needed

**Table 2:** Mixed feature library of vibration signals. IEMD, image extended empirical mode decomposition.

Feature Library	Feature	Index
Mixed-domain feature library	Time domain	Root mean square, square root amplitude, variance, standard deviation, kurtosis, waveform index, peak index, pulse index, margin index, kurtosis index
	Frequency domain	Center of gravity frequency, mean square frequency, frequency variance
	Time–frequency domain	The first eight energy entropy of the IMF (intrinsic mode function) component of IEMD decomposition

**Table 3:** Time domain characteristic index.

Time Domain Index		Calculation Formula
Dimensional indicators	Root mean square Square root amplitude Variance Standard deviation Kurtosis	$x_{rms}={\left(\frac{1}{N}\sum_{i=0}^{N-1}x_{i}{}^{2}\right)}^{1/2}$ $x_{r}={\left(\frac{1}{N}\sum_{i=0}^{N-1}{\left|x_{i}\right|}^{\frac{1}{2}}\right)}^{2}$ $\delta =\frac{1}{N}\sum_{i=0}^{N-1}{\left(x_{i}-\bar{x}\right)}^{2}$ $x_{std}={\left(\frac{1}{N}\sum_{i=1}^{N}{\left(x_{i}-\bar{x}\right)}^{2}\right)}^{1/2}$ $\beta =\frac{1}{N}\sum_{i=1}^{N}x_{i}{}^{4}$
Dimensionless index	Waveform index Peak index Pulse index Margin index Kurtosis index	$K=x_{rms}/x^{\prime }\;\left(x^{\prime }=\frac{1}{N}\sum_{i=0}^{N-1}\left|x_{i}\right|\right)$ $C=x_{p}/x_{rms}$ $I=x_{p}/x^{\prime }$ $L=x_{p}/x_{r}$ Kr=1N∑i=1Nxi4xrms4

**Table 4:** Frequency domain characteristic index.

Frequency Domain Index	Calculation Formula
Center of gravity frequency Mean square frequency Root mean square frequency Frequency variance	$FC=\frac{1}{2\pi f_{s}}\cdot \frac{\int_{0}^{\pi }\omega S\left(\omega \right)d\omega }{\int_{0}^{\pi }S\left(\omega \right)d\omega }$ $MSF=\frac{1}{4\pi^{2}f_{s}{}^{2}}\cdot \frac{\int_{0}^{\pi }\omega^{2}S\left(\omega \right)d\omega }{\int_{0}^{\pi }S\left(\omega \right)d\omega }$ $RMSF={\left(\frac{1}{4\pi^{2}f_{s}{}^{2}}\cdot \frac{\int_{0}^{\pi }\omega^{2}S\left(\omega \right)d\omega }{\int_{0}^{\pi }S\left(\omega \right)d\omega }\right)}^{\frac{1}{2}}$ $VF=\frac{1}{4\pi^{2}f_{s}{}^{2}}\cdot \frac{\int_{0}^{\pi }\left(\omega -2\pi f_{s}FC\right)S\left(\omega \right)d\omega }{\int_{0}^{\pi }S\left(\omega \right)d\omega }$

**Table 5:** Mixed feature library of stator current signals.

Feature Library	Feature	Index
Mixed-domain feature library	Time domain	Root mean square, square root amplitude, variance, standard deviation, kurtosis, waveform index, peak index, pulse index, margin index, kurtosis index
	Frequency domain	Center of gravity frequency, mean square frequency, root mean square frequency, frequency variance
	Time–frequency domain	Sample entropy *1–5*, energy entropy *H*_1_, *H*_2_, *H*_3_, *H*_4_, *H*_5_, spectral kurtosis *a*_1_, *a*_2_, *a*_3_, *a*_4_, *a*_5_

**Table 6:** Parameters in four vibration least square support vector machine (LSSVM).

LSSVM	Γ	$\sigma^{2}$	A	B
(normal, the rest)	84.2784	31.1601	−10.8211	−4.2378
(parallel misalignment, the rest)	85.8947	30	−7.4866	−1.9740
(angular misalignment, the rest)	36.0326	39.9616	−6.1128	−1.5637
(integrated misalignment, the rest)	99.4093	96.6015	−6.9786	−2.4798

**Table 7:** Basic probability assignment 1 (BPA1) of vibration LSSVM.

Sample Number	Normal	Parallel Misalignment	Angular Misalignment	Integrated Misalignment
5	0.8433	0.0314	0.0538	0.0715
44	0.0080	0.9246	0.0295	0.0379
82	0.0025	0.0070	0.8873	0.1032
130	0.0208	0.0147	0.0495	0.9150

**Table 8:** Parameters in four temperature LSSVM.

LSSVM	Γ	$\sigma^{2}$	A	B
(normal, the rest)	97.4952	89.7017	−3.0178	−0.4262
(parallel misalignment, the rest)	98.3829	30	−2.8893	0.1963
(angular misalignment, the rest)	46.3907	88.9951	−3.9974	−0.3622
(integrated misalignment, the rest)	93.6931	96.3611	−2.4749	0.3805

**Table 9:** BPA2 of temperature LSSVM.

Sample Number	Normal	Parallel Misalignment	Angular Misalignment	Integrated Misalignment
5	0.7418	0.0387	0.0228	0.1967
44	0.1314	0.7670	0.0243	0.0773
82	0.3644	0.0357	0.5549	0.0450
130	0.4365	0.0394	0.0220	0.5021

**Table 10:** Parameters in four current LSSVM.

LSSVM	Γ	$\sigma^{2}$	A	B
(normal, the rest)	35.2345	53.0811	−4.858	−0.6011
(parallel misalignment, the rest)	87.5286	30	−3.2022	−0.1165
(angular misalignment, the rest)	100	30	−3.2755	−0.2514
(integrated misalignment, the rest)	77.1503	33.2639	−3.2803	−0.2146

**Table 11:** BPA3 of current LSSVM.

Sample Number	Normal	Parallel Misalignment	Angular Misalignment	Integrated Misalignment
5	0.8635	0.0440	0.0468	0.0457
44	0.0164	0.6193	0.3159	0.0484
82	0.0170	0.1236	0.8093	0.0501
130	0.0127	0.1093	0.0521	0.8259

**Table 12:** Probability assignment of three LSSVMs and fusion.

BPA 1	BPA 2	BPA3	D–S Evidence Fusion
[0.8433, 0.0314, 0.0538, 0.0715]	[0.7418, 0.0387, 0.0228, 0.1967]	[0.8635, 0.0440, 0.0468, 0.0457]	[0.9986, 0.0001, 0.0001, 0.0012]
[0.0080, 0.9246, 0.0295, 0.0379]	[0.1314, 0.7670, 0.0243, 0.0773]	[0.0164, 0.6193, 0.3159, 0.0484]	[0.0001, 0.9991, 0.0005, 0.0003]
[0.0025, 0.0070, 0.8873, 0.1032]	[0.3644, 0.0357, 0.5549, 0.0450]	[0.0170, 0.1236, 0.8093, 0.0501]	[0.0001, 0.0001, 0.9993, 0.0005]
[0.0208, 0.0147, 0.0495, 0.9150]	[0.4365, 0.0394, 0.0220, 0.5021]	[0.0127, 0.1093, 0.0521, 0.8259]	[0.0003, 0.0002, 0.0001, 0.9994]

**Table 13:** Fusion and classification results of four test samples.

D–S Evidence Fusion	Category	Is the Classification Correct?
[0.9986, 0.0001, 0.0001, 0.0012]	Normal	Yes
[0.0001, 0.9991, 0.0005, 0.0003]	Parallel misalignment	Yes
[0.0001, 0.0001, 0.9993, 0.0005]	Angular misalignment	Yes
[0.0003, 0.0002, 0.0001, 0.9994]	Integrated misalignment	Yes

**Table 14:** Comparison of diagnostic results.

Signal Selection	Training Set Classification Accuracy	Testing Set Classification Accuracy	False Alarm Rate
Vibration signal	100% (240/240)	85.625% (137/160)	5% (2/40)
Temperature signal	90.8333% (218/240)	81.25% (130/160)	35% (14/40)
Current signal	99.5833% (239/240)	84.375% (135/160)	10% (4/40)
D–S evidence fusion	100% (240/240)	98.125% (157/160)	0% (0/40)

**Table 15:** Part of the characteristic index of the vibration signal.

Fault Type	Root Mean Square Value	Center of Gravity Frequency	IMF1 Energy Entropy	IMF2 Energy Entropy
Normal	0.0286	−118.3859	0.3671	0.0991
	0.0270	−184.0340	0.3461	0.1265
	0.0288	−308.9050	0.3678	0.1624
	0.0626	−993.2476	0.3524	0.3675
Parallel misalignment	0.0248	−272.4819	0.3678	0.1229
	0.0258	−196.5053	0.3677	0.1201
	0.0253	−286.5526	0.3668	0.1769
	0.0607	−1082.4788	0.3678	0.3455
Angular misalignment	0.0266	−166.9377	0.3488	0.1005
	0.0296	−145.9158	0.3658	0.1083
	0.0280	−232.8465	0.3620	0.1347
	0.0569	−1052.415	0.3583	0.3677
Integrated misalignment	0.0284	−261.2838	0.3544	0.1615
	0.0342	−334.0774	0.3621	0.2021
	0.0311	−388.9565	0.3675	0.2138
	0.0670	−1138.6520	0.3603	0.3670

**Table 16:** Partial characteristic index of current signal.

Fault Type	Root Mean Square Value	Center of Gravity Frequency	Energy Entropy1	Sample Entropy1
Normal	2.4944	−0.0571	0.0001	0.6859
	2.4952	−0.0741	0.0003	0.8285
	3.5641	−0.1023	0.0008	0.9624
Parallel misalignment	2.4948	−0.1238	0.0001	0.6099
	2.5293	−0.2608	0.0004	0.7046
	2.7607	−0.4788	0.0015	0.9455
Angular misalignment	2.4990	−0.1062	0.0002	1.0642
	2.6908	−0.2794	0.0008	1.1659
	3.0569	−0.4415	0.0016	1.3677
Integrated misalignment	2.5051	−0.0524	0.0003	1.2666
	2.8986	−0.3861	0.0009	1.6857
	3.2670	−0.6520	0.0023	1.7670

**Table 17:** Basic probability assignment of two kinds signals and probability after fusion.

Probability Value	Normal	Parallel Misalignment	Angular Misalignment	Integrated Misalignment
Vibration signal	0.5161 0.1770	0.1663 0.1927	0.1510 0.4194	0.1666 0.2109
Current signal	0.8209 0.7705	0.0737 0.0566	0.0181 0.1063	0.0873 0.0666
Fusion signal	0.9348	0.0281	0.0050	0.0321
	0.6623	0.0530	0.2164	0.0683
